# A comparison of urinary bladder weight in male and female mice across five models of diabetes and obesity

**DOI:** 10.3389/fphar.2023.1118730

**Published:** 2023-02-20

**Authors:** Betül R. Erdogan, Martina B. Michel, Jan Matthes, Tamara R. Castañeda, Urs Christen, Ebru Arioglu-Inan, Martin C. Michel, Andrea Pautz

**Affiliations:** ^1^ Department of Pharmacology, Faculty of Pharmacy, Izmir Katip Celebi University, Izmir, Türkiye; ^2^ Department of Pharmacology, Johannes Gutenberg University, Mainz, Germany; ^3^ Centre of Pharmacology, University Medical Center, University of Cologne, Cologne, Germany; ^4^ Sanofi Research and Development, Frankfurt, Germany; ^5^ Pharmazentrum, Goethe University, Frankfurt, Germany; ^6^ Department of Pharmacology, Faculty of Pharmacy, Ankara University, Ankara, Türkiye

**Keywords:** mouse, diabetes, obesity, bladder, hypertrophy, sex difference

## Abstract

**Introduction:** Diabetes often leads to lower urinary tract dysfunction. The most frequently assessed parameter of urinary bladder dysfunction in animal models of diabetes is an enlargement of the bladder, which is consistently observed in type 1 and less consistently in type 2 diabetes. The vast majority of studies on bladder weight in animal models of diabetes and obesity has been performed in males, and no studies have directly compared this outcome parameter between sexes.

**Methods:** Therefore, we have compared bladder weight and bladder/body weight ratio in five mouse models of obesity and diabetes (RIP-LCMV, db/db, ob/ob (two studies), insulin receptor substrate 2 (IRS2) knock-out mice and mice on a high-fat diet; pre-specified secondary analysis of a previously reported study).

**Results:** In a pooled analysis of the control groups of all studies, females exhibited slightly lower glucose levels, lower body weight, and lower bladder weight, but bladder/body weight ratio was similar in both sexes (0.957 vs. 0.986 mg/g, mean difference 0.029 [−0.06; 0.118]). Among the six diabetic/obese groups, bladder/body weight ratio was similar in both sexes in three but smaller in female mice in three other groups. The mRNA expression of a panel of genes implied in the pathophysiology of bladder enlargement and/or fibrosis and inflammation did not differ systematically between sexes.

**Conclusions:** We conclude that sex differences in diabetes/obesity-associated bladder enlargement may be model dependent.

## 1 Introduction

Lower urinary tract (LUT) dysfunction refers a functional impairment of the LUT organs including bladder, urethra and prostate (in men). LUT dysfunction in general and urinary bladder dysfunction in particular belong to the most frequent complications of diabetes and are reported to occur in 80% and 50%, respectively, of diabetic patients (Daneshgari and Moore, 2006). In contrast to cardiovascular, renal and ocular complications, LUT dysfunction typically does not lead to mortality or major morbidity but has major adverse effects on the quality of life of the afflicted patients (Irwin et al., 2008; Benner et al., 2009) and their partners (Mitroupoulos et al., 2002) and is associated with increased emergency room visits and hospitalizations, and loss of work productivity (Kannan et al., 2009).

Diabetes manifests differently in male and female patients for various parameters including post-prandial glucose levels (Diaf et al., 2014), glucose tolerance (Mauvais-Jarvis, 2017), and insulin secretion and action (Basu et al., 2017). Some of these differences were also found in euglycemic, non-obese subjects (Chan et al., 2019). Sex differences have also been reported in animal models of diabetes, e.g., for mitochondrial content and oxidative phosphorylation (Schneider et al., 2020) and oxidative stress (Díaz et al., 2019) or genetic associations (Wagener et al., 2006). Moreover, there are sex and gender differences in the epidemiology of diabetes and its risk factors (Seghieri et al., 2022).

Various animal models of type 1 and type 2 diabetes (T1DM and T2DM, respectively) and of obesity exhibit LUT dysfunction in general and/or bladder dysfunction in particular. Specifically, more than 150 studies in animal models report on urinary bladder weight (Arioglu-Inan et al., 2018; Ellenbroek et al., 2018; Yesilyurt et al., 2022). They demonstrate that bladder enlargement is a universal feature of rodent models of T1DM, whereas it occurs less consistently in those of T2DM. This mirrors the prevalence of bladder dysfunction in 43%–87% of T1DM patients and 25% of T2DM patients (Kempler et al., 2011). However, there is no consistent relationship between severity of diabetes assessed as blood glucose levels and presence or extent of bladder enlargement (Ellenbroek et al., 2018; Yesilyurt et al., 2022).

Bladder enlargement occurs in many animal models and patient populations including animals with bladder outlet obstruction, bladder denervation or treatment with osmotic diuretics or in patients with overactive bladder syndrome. Bladder enlargement is associated with bladder dysfunction in all settings where it has been tested, but cause-effect relationships between LUT dysfunction and bladder enlargement have not been established robustly. Various pathophysiological factors have been proposed to be involved in bladder enlargement and dysfunction in diabetic and euglycemic animals, including nerve growth factor (NGF) and its receptor tropomyosin receptor kinase A (trk A) (Ochodnicky et al., 2011) and angiotensin II type 1 and 2 receptors (AT_1_ and AT_2_ receptor, respectively (Yamada et al., 2009). Moreover, an enlargement of the urinary bladder and other tissues often involves fibrosis (Ochodnicky et al., 2011; Shen et al., 2015) and/or inflammation indicated by markers such as monocyte chemoattractant protein-1 (MCP-1) (Sukumaran et al., 2012) or interleukin-6 (IL-6) (Shen et al., 2015).

A leading theory of LUT dysfunction in general and bladder dysfunction in particular proposes that it occurs secondary to chronic pelvic hypoperfusion/ischemia that occurs in relation to atherosclerosis of blood vessels supplying the LUT (Michel et al., 2015; Thurmond et al., 2016). Metabolic syndrome and diabetes are known risk factors of atherosclerosis. While the relative roles of risk factors for atherosclerosis appears to have shifted in recent years, sex and gender are considered underappreciated risk factors (Libby, 2021). The vast majority of studies of bladder weight in animal models of diabetes and obesity is based on male animals and only few studies have reported on female animals (Arioglu-Inan et al., 2018; Ellenbroek et al., 2018). An indirect comparison across studies in the streptozotocin injection model of T1DM found that bladder enlargement was numerically greater in 12 cohorts of female than in 59 cohorts of male rats (Arioglu-Inan et al., 2018), but these data are difficult to interpret due to the indirect comparison and the large inter-study variability within each sex. Although bladder enlargement in models unrelated to diabetes such as Bartter’s mice has been reported to be present in male but not female animals (Kim et al., 2018), to the best of our knowledge, no studies directly comparing bladder weight changes between sexes in animal models of diabetes and obesity exist.

We have recently reported bladder weight data from 16 studies across nine distinct rodent models of diabetes and obesity (Yesilyurt et al., 2022). Five of these studies had included both male and female mice, one of them in two models. Therefore, we have performed a pre-planned direct comparison of bladder weight and bladder/body weight ratio in both sexes for these five studies and related those findings to possible difference in glucose levels and body weight. One of these models, IRS-2 knock-out mice, exhibits a known sexual dimorphism regarding extent of glucose elevation (Kubota et al., 2000; Masaki et al., 2004) that is associated with a differential survival (Withers et al., 1998). Using three of these studies as example, we also have compared mRNA expression of various genes related to fibrosis/inflammation or proposed to be involved in the pathophysiology of diabetic bladder dysfunction and enlargement between sexes.

## 2 Materials and methods

### 2.1 Animal models

The present data represent a prespecified analysis of findings from a previously published report for the studies that had included both sexes (Yesilyurt et al., 2022). Specifically, five models were included, one of which (ob/ob mice) being used in two of the studies. All studies were conducted in the lab of one of the authors as identified by the applicable institution. Details of each model are included in our previous report including information on housing and husbandry according to the ARRIVE 2.0 guidelines (Percie du Sert et al., 2020). Blood sampling for glucose measurement was made at time of sacrifice in a fed state in the morning.

Briefly, one study used a model of T1DM, RIP-LCMV mice from the lab of UC, which is based on expression of the glycoprotein of the lymphocytic choriomeningitis virus under control of the rat insulin promoter in the beta-cells of the islets of Langerhans (Oldstone et al., 1991). These mice have been backcrossed to a C57BL/6J background for more than 30 years. It had been approved by the Ethics Committee of the State Ministry of Agriculture, Nutrition and Forestry, State of Hessen, Germany (V54-19c20/15-FU-1192 and V54-19c20/15-FU-1213). 8-10-week-old mice were infected with LMCV and killed 6–8-week after diabetes had developed by cervical dislocation isoflurane anesthesia; age-matched RIP-LMCV-GP mice were used as control.

The second study used db/db mice, a genetic model of T2DM based on a deficient leptin receptor due to a point mutation in the corresponding gene, and wild-type C57BL/6J mice as control from the lab of TRC. Based on the applicable German animal protection law, no animal permit was required for tissue harvesting not linked to experimental procedures; instead, an internal permit was applicable and the report of the number of animals used. Mice at 12-week-old were sacrificed by cervical dislocation under isoflurane anesthesia.

ob/ob mice, a model of T2DM based on a recessive mutation in the leptin gene, were tested in comparison to wild-type C57BL/6J mice in two studies, one being the above study also involving db/db mice (in the lab of TRC, labelled as Hoechst), the other a dedicated study (in the lab of JM, labelled as Cologne), which had been approved by the responsible federal state authority (Landesamt für Natur-, Umwelt-und Verbraucherschutz Nordrhein-Westfalen; 84-02.04.2016.A049 and 84-02.04.2016.A422). Age matched 12-week-old ob/ob and control mice were used in the Hoechst study under the same study protocol as in db/db mice study. 21–56-week-old ob/ob and control mice (ob/ob male and female 25.5 ± 3.1 vs. 32.1 ± 14.1 weeks, respectively; ob/ob control male and female 28.2 ± 0.4 vs. 40.4 ± 14.5 weeks, respectively) were used in the Cologne study. Mice were sacrificed by cervical dislocation.

The fourth study used insulin receptor substrate 2 (IRS2) knock-out mice, another T2DM model exhibiting impairment of peripheral insulin signaling and pancreatic beta-cell function (Oliveira et al., 2014) in comparison to C57BL/6J mice from the lab of JM; it had been approved under the same permit as the Cologne ob/ob mouse study. 16–61-week-old mice (male and female IRS2 KO 31.2 ± 11.1 vs. 35.7 vs. 14.2 weeks, respectively; IRS2 controls 25.0 ± 5.1 vs. 38.9 vs. 14.9 weeks, respectively) were used and the same protocol was applied as the Cologne ob/ob mouse study.

The fifth study compared high-fat diet to standard diet in C57BL/6N mice, a model that does not exhibit overt diabetes but is obese and exhibits modestly elevated blood glucose and reduced insulin sensitivity. It was performed in the lab of TRC and had been approved by the Ethics Committee of the State Ministry of Agriculture, Nutrition and Forestry, State of Hessen, Germany (the V54-19 c 20/15-FH/Anz. 1024 and T4-12.A1). 12-week-old C57BL/6N control and 24-week-old HFD mice were used this study. Mice were by an isoflurane overdose and euthanized by cervical dislocation. Bladder tissues were excised and weighed, and blood glucose levels were measured.

### 2.2 mRNA analysis

The bladder tissue from the db/db, ob/ob (both studies) and IRS2 knock-out mice and their matched controls were included in the mRNA analysis. They had been selected as examples based on tissue availability. Total RNA was prepared from whole bladder by the acid guanidinium thiocyanate (GIT)-phenol-chloroform extraction method (Chomczynski and Sacchi, 1987). In brief, urinary bladder samples had been macroscopically freed from surrounding connective tissue and placed in liquid nitrogen immediately after harvesting and then transferred to a −70°C or colder freezer for storage. The bladder was split into three pieces, each homogenized in GIT buffer using a steel bead in the TissueLyzer (Qiagen, Hilden, Germany) for 5 min at 50 Hz. Subsequently, the samples were centrifuged at 900 g for 5 min at 4°C. The supernatant was transferred into a new vial, RNA isolation was performed by phenol-chloroform extraction method (Chomczynski and Sacchi, 1987), and the material from the three pieces was pooled. To eliminate genomic DNA the samples were subjected to a DNAse digestion according to manufacturer instructions (DNAse I recombinant kit, catalog number 04716728001, Roche, Basel, Switzerland) followed by another phenol extraction. Afterwards, RNA concentration was determined using the spectrophotometer NanoDrop-2000© (PeqLab, Erlangen, Germany). The cDNA reaction was performed with the High-Capacity cDNA Reverse Transcription Kit (Applied Biosystems/Thermo Fisher, Langenselbold, Germany) using 500 ng RNA and Hexa-N-Oligonucleotides according to the protocol of the manufacturer. Gene expression in samples was quantified in a two-step real-time RT-PCR using the Blue S’Green qPCR Kit (Biozym, Hessisch Oldendorf, Germany) according to the recommendation of the manufacturer. Oligonucleotides used for quantitative real-time RT-PCR (qRT-PCR) were designed with the Primer 3Plus software tool and are shown in the Online Supplementary Table S1. Specific mRNA expression was measured using the qRT-PCR method and normalized to GAPDH and β-actin mRNA expression. To calculate the relative mRNA expression the 2(−^ΔΔ^C_T_) method was used (Livak and Schmittgen, 2001). As we used a SYBRgreen PCR system, only primers and samples with one distinct melting curve were analyzed. The double normalization for expression of GAPDH and β-actin within a sample was justified because their expression did not differ between sexes in the pooled data from all control mice (mean difference with CI: GADPH 0.07 [−0.61; 0.75], β-actin 0.17 [−0.87; 1.21]; Supplementary Figure S1). Thus, expression of a target gene within a sample was normalized to GAPDH and β-actin and the mean normalized expression was used. We then set the mean of all samples from male mice within a study as 100% and expressed each male and female sample as % of that mean.

### 2.3 Data analysis

Blood glucose levels, body weight, and bladder weight were measured, and bladder/body weight ratio calculated for each mouse. Our comparisons were limited to those of both sexes within a group and study because intergroup comparisons of control and diabetic/obese with corresponding control mice were reported previously (Yesilyurt et al., 2022). A sex comparison of pooled data from the control euglycemic groups of all studies was performed as internal validation.

Based on the exploratory character of our study and in line with recent recommendations of leading statisticians (Amrhein et al., 2019; Michel et al., 2020), we did not perform hypothesis-testing statistical analyses. Rather we focus on effect sizes for sex differences with their 95% confidence intervals (CI) as calculated from unpaired, two-tailed t-tests using GraphPad Prism version 9.3 or higher (GraphPad, Los Angeles, CA, United States). We arbitrarily assumed that a sex difference of >10% could be considered as biologically relevant. Based on previous recommendation (Erdogan et al., 2020), we used the same y-axis spread for all graphs showing a parameter to avoid bias in reporting.

## 3 Results

### 3.1 Model validation

**FIGURE 1:**
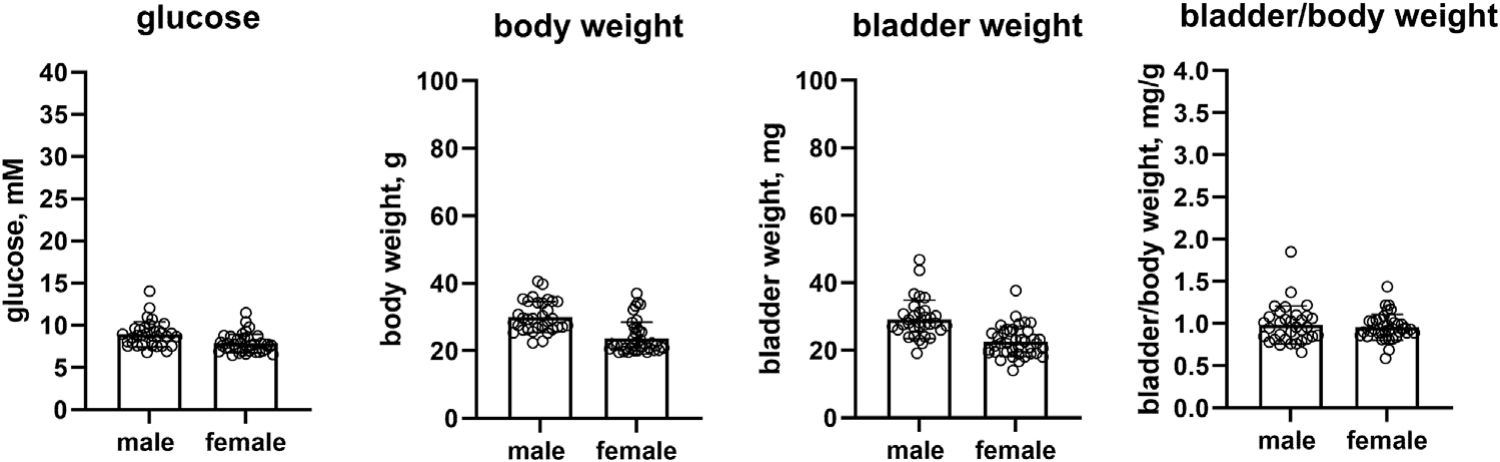
Glucose, body weight, bladder weight and bladder/body weight ratio in the pooled control mice of all studies. Data are means ± SD with each animal shown as circle. Data from control groups of individual studies are shown in online Supplementary Figures S2–S6.

In a pooled analyses of the control groups of all studies, female as compared to male mice had lower glucose levels (7.86 vs. 8.92 mM, mean difference 1.06 mM [95% CI 0.44; 1.68]), a substantially smaller body weight (23.8 vs. 30.1 g, mean difference 6.2 g [4.0; 8.4]) and bladder weight (22.6 vs. 29.2 mg, mean difference 6.6 mg [4.2; 9.0]) whereas bladder/body weight ratio was similar in both sexes (0.957 vs. 0.986 mg/g, mean difference 0.029 [−0.06; 0.118]) (n = 39 and 33, Figure 1). Data on the controls within each study are shown in the Supplementary Figures S2–S6.

### 3.2 Physiological parameters

#### 3.2.1 RIP-LCMV mice

**FIGURE 2:**
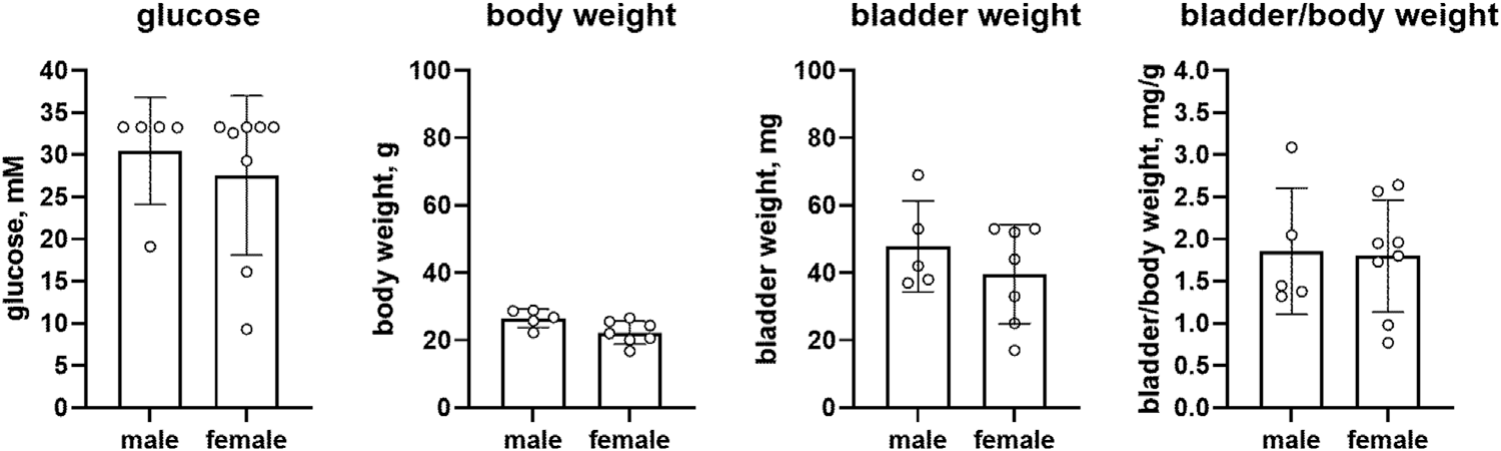
Glucose, body weight, bladder weight and bladder/body weight ratio in RIP-LCMV mice. Data are means ± SD with each animal shown as circle.

Female RIP-LCMV mice as compared to male mice had comparable glucose levels (27.6 vs. 30.4 mM, mean difference 2.9 mM [95% CI −7.7; 13.5]) but a lower body weight (22.3 vs. 26.5 g, mean difference 4.2 g [0.1; 8.3]). While bladder weight differed by >10% between sexes (39.6 vs. 47.8 mg, mean difference 8.2 mg [−10.3; 26.8]), these data are difficult to interpret based on large CI. Bladder/body weight was similar in both groups (1.800 vs. 1.858, mean difference 0.058 [−0.815; 0.931], n = 8 and 5, respectively; Figure 2).

#### 3.2.2 db/db mice

**FIGURE 3:**
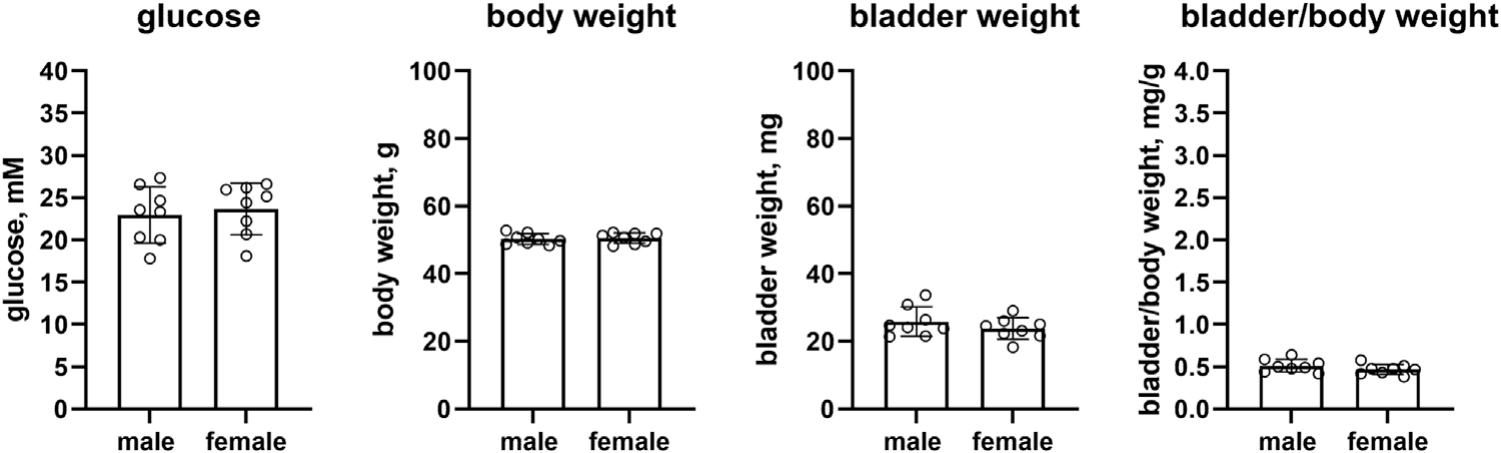
Glucose, body weight, bladder weight and bladder/body weight ratio in db/db mice. Data are means ± SD with each animal shown as circle.

Female db/db mice as compared to male mice had similar glucose levels (23.7 vs. 22.9 mM, mean difference −0.7 mM [95% CI −4.1; 2.7]), body weight (50.6 vs. 50.3 g, mean difference: −0.3 g [95% CI −2.0; 1.4]), bladder weight (23.7 vs. 25.8 mg, mean difference 2.1 mg [−2.0; 6.2]) and bladder/body weight (0.469 vs. 0.513, mean difference 0.044 [−0.029; 0.117], n = 8 each, Figure 3).

#### 3.2.3 ob/ob mice (hoechst)

**FIGURE 4:**
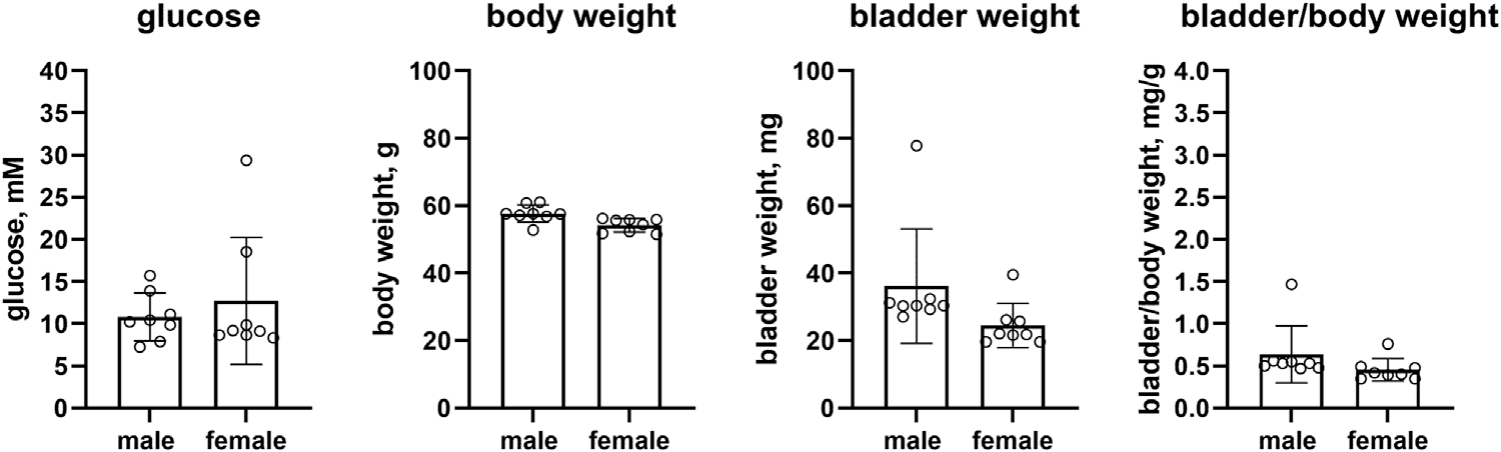
Glucose, body weight, bladder weight and bladder/body weight ratio in ob/ob mice (Hoechst). Data are means ± SD with each animal shown as circle.

Female ob/ob mice as compared to male mice had numerically higher glucose levels, but this was inconclusive due to a wide CI (12.7 vs. 10.8 mM, mean difference −1.9 mM [95% CI −8.0; 4.2]). While body weight was similar in both sexes (54.2 vs. 57.7 g, mean difference 3.5 g [95% CI 1.0; 5.9]), bladder weight was numerically lower in female ob/ob mice albeit with a large CI (24.5 vs. 36.1 mg, mean difference 11.6 mg [−2.1; 25.4]). This was similarly observed for bladder/body weight (0.455 vs. 0.636, mean difference 0.181 [−0.095; 0.457], n = 8 each, Figure 4).

#### 3.2.4 ob/ob mice (cologne)

**FIGURE 5:**
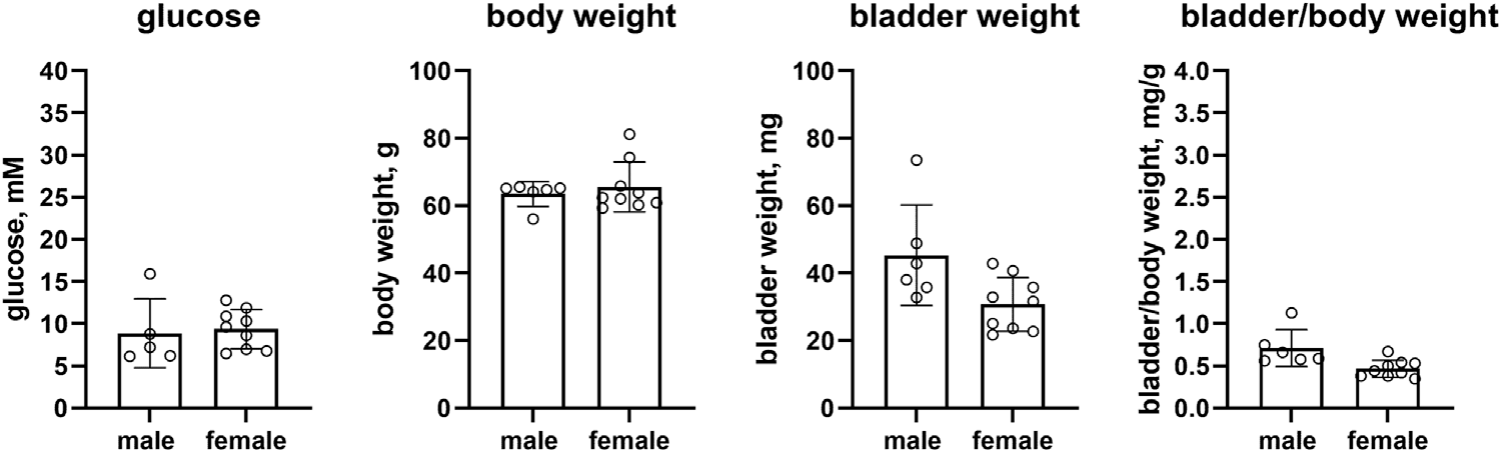
Glucose, body weight, bladder weight and bladder/body weight ratio in ob/ob mice (Cologne). Data are means ± SD with each animal shown as circle.

In the second study of this model, female ob/ob mice as compared to male mice had similar glucose levels (9.37 vs. 8.86 mM, mean difference −0.51 mM [95% CI −4.18; 3.16]) and body weight (65.5 vs. 63.4 g, mean difference −2.1 g [95% CI −9.2; 5.0]), whereas bladder weight (30.8 vs. 45.3 mg, mean difference 14.6 mg [1.8; 27.3]) and bladder/body weight (0.468 vs. 0.712, mean difference 0.244 [0.066; 0.422]) were lower in the female ob/ob mice (n = 9 and 6, respectively, Figure 5).

#### 3.2.5 IRS2 knock-out mice

**FIGURE 6:**
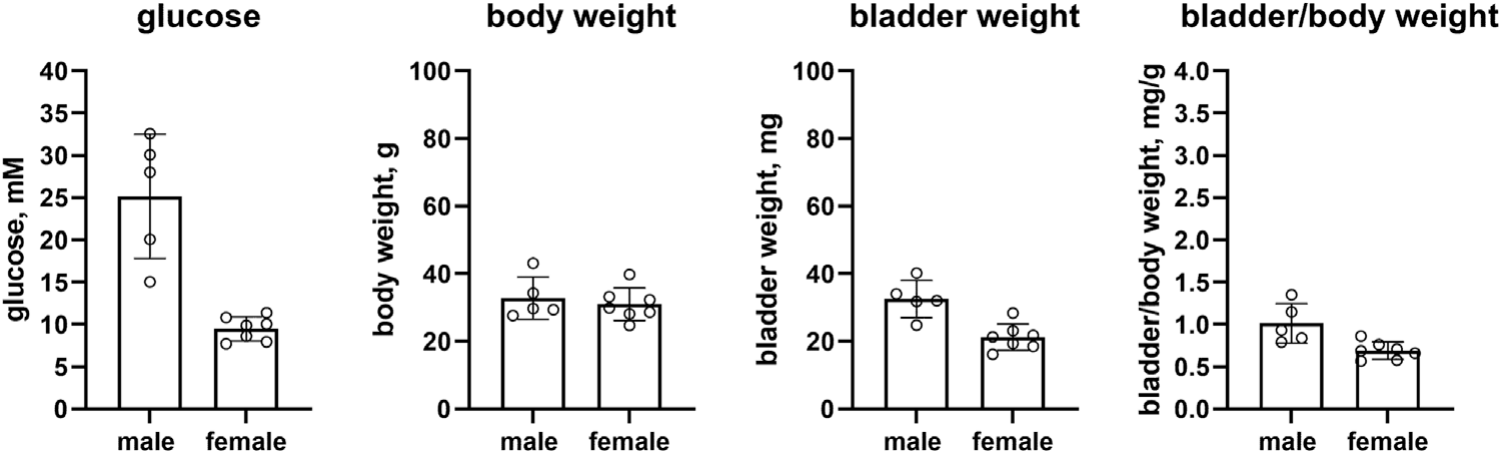
Glucose, body weight, bladder weight and bladder/body weight ratio in IRS2 knock-out mice. Data are means ± SD with each animal shown as circle.

Confirming previous reports, female as compared to male IRS2 knock-out mice had markedly lower glucose levels [9.5 vs. 25.2 mM, mean difference 15.7 mM (95% CI 9.5; 21.9)] but a comparable body weight (30.9 vs. 32.8 g, mean difference 1.9 g [95% CI −5.2; 9.0]). We extend these findings by reporting that bladder weight (21.2 vs. 32.6 mg, mean difference 11.3 mg [5.3; 17.3]) and bladder/body weight [0.690 vs. 1.012, mean difference 0.322 (0.104; 0.541)] of female mice were smaller in comparison with male mice (n = 7 and 5, respectively, Figure 6).

#### 3.2.6 Mice on high-fat diet

**FIGURE 7:**
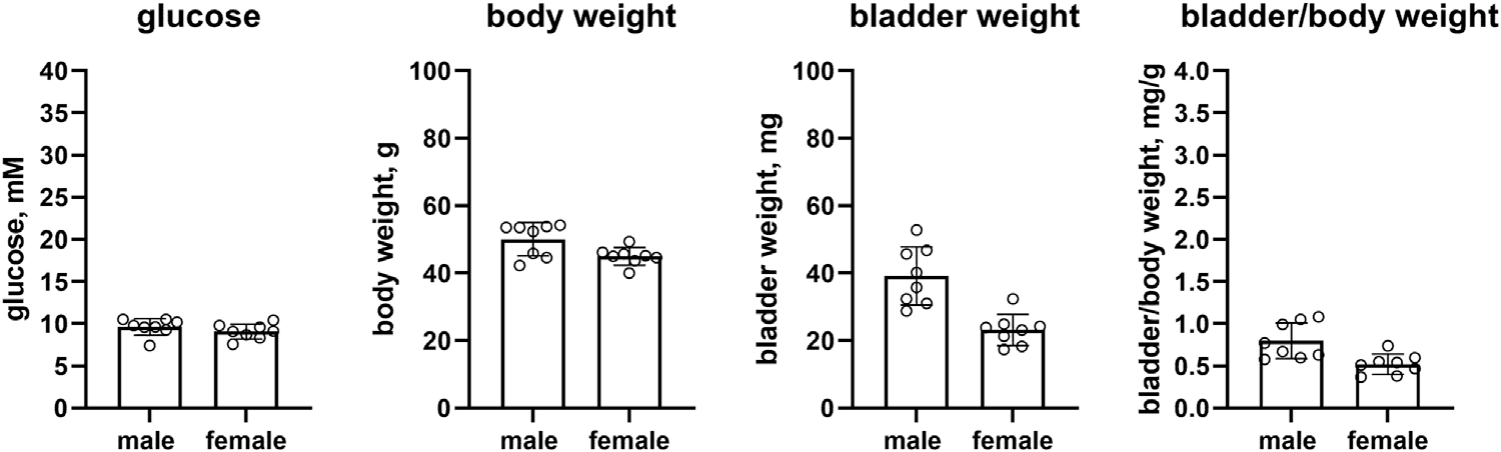
Glucose, body weight, bladder weight and bladder/body weight ratio in HFD mice. Data are means ± SD with each animal shown as circle.

Female as compared to male mice on a HFD had similar glucose levels (9.1 vs. 9.6 mM mean difference 0.5 mM [95% CI −0.5; 1.5]), and a slightly lower body weight (45.0 vs. 50.0 g, mean difference 5.1 g [CI 0.8; 9.3]). Bladder weight (23.2 vs. 39.2 mg, mean difference 16 mg [8.6; 23.4]) and bladder/body weight (0.520 vs. 0.796, mean difference 0.276 [0.092; 0.460]) of female mice were lower than in male mice (n = 8 each, Figure 7).

### 3.3 mRNA analysis

**FIGURE 8:**
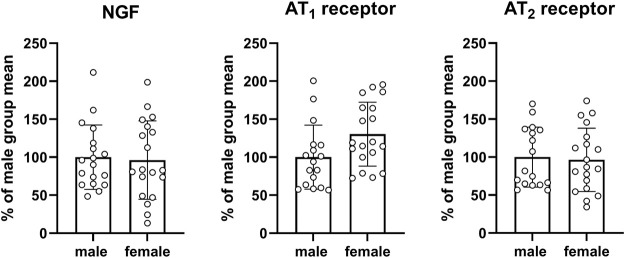
mRNA expression of genes proposed to be involved in the pathophysiology of bladder enlargement. Data are means ± SD from the pooled control groups with each animal shown as circle.

**FIGURE 9:**
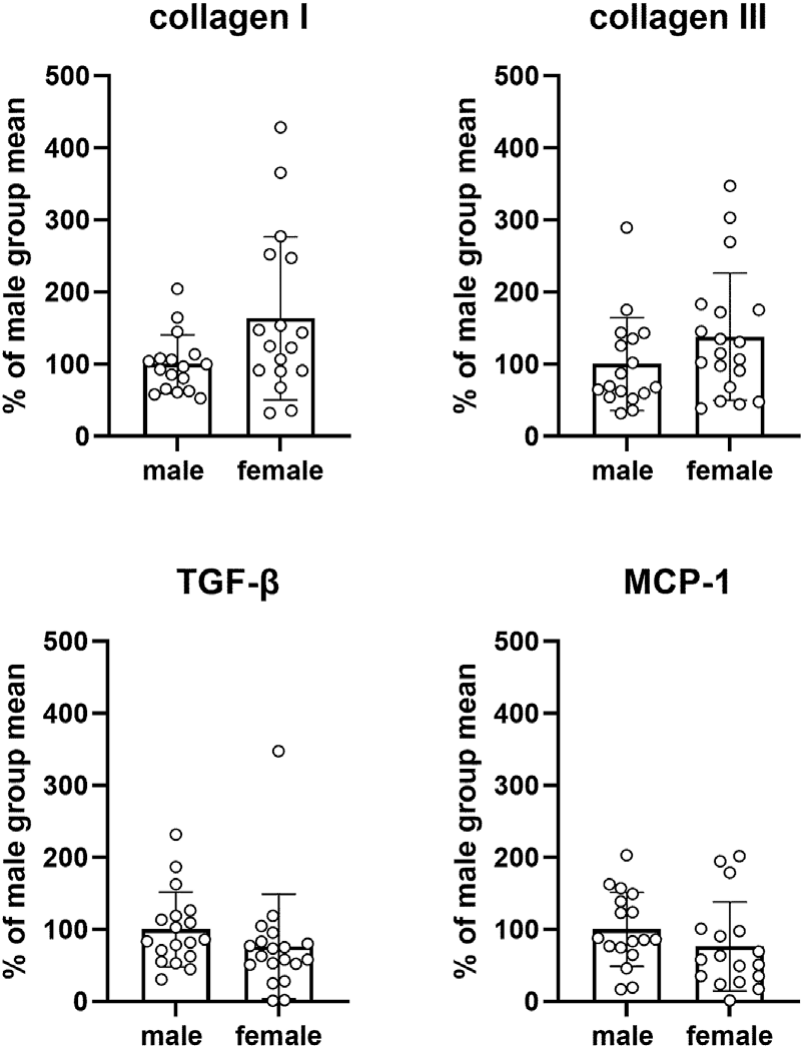
mRNA expression of genes involved in fibrosis and/or inflammation. Data are means ± SD from the pooled control groups with each animal shown as circle.

An exploratory analysis was performed for mRNA expression of various genes proposed to be involved in the pathophysiology of bladder enlargement (Figure 8) or in fibrosis/inflammation (Figure 9). Two of our target genes, trk A and IL-6, exhibited a very low expression (i.e., C_T_ values close to water control) across control and diabetic groups and, therefore, were not evaluated quantitatively. We here show data for all other target gene expressions from the pooled control groups of the three studies. Those from individual control groups and from diabetic/obese groups are shown as Supplementary Figures S7–S20 but their interpretation may be difficult because of limited sample sizes relative to parameter variability.

Three target genes proposed to be involved in the pathophysiology of bladder enlargement were studied, i.e., NGF and the two subtypes of angiotensin II receptor subtypes AT_1_ and AT_2_. Compared to males, expression in females did not differ substantially for NGF (mean difference −3.7% [−35.4; 27.9]) or the AT_2_ receptor (−3.6% [−31.1: 24.0]) but was greater for the AT_1_ receptor (30.3% [1.7; 58.9]; Figure 8) with the latter being difficult to interpret based on a large CI. Similarly, no consistent and/or major differences were found within each study in the control groups or in the diabetic/obese groups (see Supplementary Figures S7, S9, S11, S13, S15, S17, and S19).

Four target genes involved in bladder fibrosis and/or inflammation were studied, i.e., collagen I and III, TGF-β and MCP-1. Compared to males, expression in females was numerically greater for collagen I and III and numerically smaller for MCP-1 and TGF-β, but CI were wide in all cases (collagen I: 63.4% [4.0; 122.7], collagen III: 37.9% [−14.9; 90.7], MCP-1: −23.7% [−63.5; 16.0], TGF-β: −23.8% [−66.2; 18.6]; Figure 9). Similarly, no consistent and/or major differences were found within each study in the control groups or in the diabetic/obese groups (see Supplementary Figures S8, S10, S12, S14, S16, S18, and S20).

## 4 Discussion

The physiological regulation of bladder function appears similar in both sexes in experimental animals (Michel and Barendrecht, 2008). For instance, contractile responses of isolated bladder strips to field stimulation were similar in male and female rats (Longhurst and Levendusky, 2000) and pigs (Dambros et al., 2004). The contractile responses to the muscarinic receptor agonist carbachol, mimicking the endogenous parasympathetic transmitter acetylcholine, were also similar across sexes in rats (Longhurst and Levendusky, 2000; Kories et al., 2003), mice (Choppin, 2002), cats (An et al., 2002), and humans (Kories et al., 2003). Responses of isolated bladder strips in relaxation to β-adrenoceptor agonists regarding sex showed small to non-existent differences in rats, rabbits or humans (Michel and Barendrecht, 2008).

Diabetes manifests differently in males and females for various parameters (Van Liew et al., 1993; Wagener et al., 2006; Diaf et al., 2014; Basu et al., 2017; Mauvais-Jarvis, 2017; Díaz et al., 2019; Schneider et al., 2020; Seghieri et al., 2022). Notably, this includes glucose levels in one of the diabetic models studied here, IRS2 knock-out mice (Kubota et al., 2000; Masaki et al., 2004). However, to the best of our knowledge, no previous study has compared diabetes/obesity-associated bladder enlargement between sexes within a study. Therefore, we have analyzed five studies on five mouse models (in total six data sets because one model, ob/ob mice, was assessed in two studies). These rodent models represent the full spectrum of T1DM, T2DM and obesity without overt diabetes and only moderately elevated glucose levels. As some animal models of T2DM and obesity do and some do not develop bladder enlargement (Ellenbroek et al., 2018; Yesilyurt et al., 2022), we have included models with and without bladder enlargement in our analyses.

### 4.1 Critique of methods

As discussed in the primary paper reporting on glucose levels, body weight, bladder weight and bladder/body weight ratio in 16 studies involving nine distinct animal models (Yesilyurt et al., 2022), we have used data from studies that were not designed to study bladder weight. Rather we have collected data from ongoing studies designed for other purposes, which is a weakness and a strength of the current analyses. The weakness is that the underlying studies were neither designed nor powered to assess bladder weight and possible differences between euglycemic and hyperglycemic or between male and female mice. The main strength is that a comparison of so many models and studies for the purpose of a sex comparison would have raised ethical and budgetary concerns. The 3 R principles of animal research place an ethical mandate to replace, reduce and refine animal research to limit it to what is necessary to answer relevant biological questions (Sneddon et al., 2017). Because it is presently impossible to avoid animal studies to investigate a role of diabetes in the regulation of bladder size and function, reducing animal numbers becomes the prime ethical mandate in such studies. By limiting ourselves to using data from ongoing studies designed for other purposes, we have kept the number of animals exposed and killed for our scientific question to 0, which is the maximally possible reduction.

The two studies from Cologne did not apply age-matching, but we have shown previously that bladder weight was not correlated to age in these cohorts, i.e., had squared correlation coefficients (r^2^) of 0.0080 and 0.0283 (Yesilyurt et al., 2022), i.e., is unlikely to have distorted the present sex comparison. Therefore, we feel that such secondary use has a positive strength/weakness balance, particularly because our studies are designed as exploratory, and not as hypothesis testing.

Female animals are vastly underrepresented in the literature on bladder morphology and function in diabetes (Arioglu-Inan et al., 2018; Ellenbroek et al., 2018), which is similar to many other disease areas. Including male and female animals in every study and keeping it powered not only for the main biological comparison but also for a comparison of sexes creates challenges both on resource utilization and ethics of animal use (Erdogan and Michel, 2020). Therefore, our overall research program on diabetes and the urinary bladder was designed to address this in two ways. Firstly, most of our dedicated studies are based on female animals only to improve the balance of sex representation in the literature (Yesilyurt et al., 2021; Arioglu-Inan et al., 2022). Second, we had planned to compare data from both sexes for all studies in which animals of both sexes had been included. This was the case in five studies involving five distinct mouse models of diabetes and obesity (Yesilyurt et al., 2022), which are analyzed here.

Enlargement/hypertrophy at the organ level is often assessed relative to denominators such as body weight or, particularly in mice, tibia length (Gao et al., 2022). For instance, the latter is standard when exploring cardiac hypertrophy in disease and upon treatment in mice (Dewenter et al., 2017; Tomimatsu et al., 2022). Given that most animal models of T1DM involve a lowered body weight and many of T2DM an increased body weight, normalization for body weight may create a bias in the comparison of euglycemic and hyperglycemic animals based on the choice of denominator. This would indicate that normalization for tibia length may be more appropriate in animal studies of diabetes, but none of the studies obtained for our analyses had done that. Therefore, we had chosen to focus on bladder weight without adjustment for body weight for the comparison of control and diabetic animals, particularly when comparing data from multiple models in our previous work on diabetes-associated bladder enlargement (Yesilyurt et al., 2022). In contrast, data on bladder/body weight were only reported as secondary outcome parameter. For the present analyses we have elected to primarily focus on bladder/body weight ratio as outcome parameter because all comparisons were made within a model and group and because body weight differences between sexes are typical for mice (see next section).

### 4.2 Sex differences in control mice

The body weight of healthy female mice is lower than that of male mice, and animal suppliers provide charts of the sex-specific development of body weight across the lifespan of mice, including data from C57BL/6J and C57BL/6N mice, the two strains used as control in our analyses (e.g., https://www.jax.org/jax-mice-and-services/strain-data-sheet-pages/body-weight-chart-000664). The smaller body weight of pooled female as compared to male control mice therefore validates our assessments (Figure 1).

Fasting blood glucose levels are lower in women than in men, and such differences are not explained by anthropomorphic measures (Faerch et al., 2010). Our pooled data from control mice of the five studies similarly found that females had about 1 mM lower glucose levels than males (Figure 1). However, data on blood glucose levels in rodents may be more complex. For instance, glucose levels have been reported to decline with age in male but to increase in female Fischer 344 rats (Van Liew et al., 1993). Of note, our pooled data are based on 33 and 39 mice per sex, whereas most other studies had used much lower animal numbers, which may be prone to inconsistent results based on chance alone (Halsey et al., 2015).

Our euglycemic female mice had a substantially lower bladder weight but such differences disappeared after correction for the similarly lower body weight. This is in line with previous observations in euglycemic rats (Frazier et al., 2006), although others found that the difference in body weight was greater than that in bladder weight leading to an increased bladder/body weight ratio in female rats (Longhurst and Levendusky, 2000). Others reported that voiding pattern differed between sexes across multiple rat strains (Patra et al., 2007).

### 4.3 Physiological sex differences in diabetic/obese mice

The mouse models of diabetes/obesity used here did not exhibit intra-study sex differences in glucose levels, except for the IRS2 knock-out mice, where this is a known sexual dimorphism (Kubota et al., 2000; Masaki et al., 2004). Our data in the pooled control mice confirm that females have a lower body weight than males. However, this was found within the diabetic/obese models only for RIP-LCMV mice and those on an HFD. There are two possible and not mutually exclusive explanations for this observation. Firstly, it is possible that a body weight difference of about 20% as observed in 33–39 control mice per sex does not show up consistently in the smaller groups of individual models of diabetes/obesity based on chance alone (Halsey et al., 2015). Second, it is also possible that the smaller body weight of female euglycemic mice is not conserved under conditions of diabetes/obesity. We consider that the second possibility is at least partly applicable because none of the diabetic/obese groups exhibited a numeric body weight difference even close to the 20% observed in control mice.

A previous study has reported that male Bartter’s mice exhibit urinary bladder enlargement whereas females do not (Kim et al., 2018). On the other hand, an indirect comparison between studies in the streptozotocin injection-based model of T1DM in rats found that bladder enlargement was numerically greater in female than in male animals—albeit with large inter-study variability in both sexes (Arioglu-Inan et al., 2018). These findings were the primary motivation for the present analyses which, to the best of our knowledge, represent the first direct comparison of diabetes/obesity-associated bladder enlargement between sexes. As discussed in Section 4.1, we primarily have based this comparison on bladder/body weight ratio. Our data are heterogeneous across models. Bladder/body weight ratio was similar in both sexes in RIP-LCMV and in db/db mice. This was also the case in one of the ob/ob mice studies, whereas females had an about 35% lower ratio in the other ob/ob mice study. A sex difference of similar magnitude was also observed in the IRS2 knock-out mice, a model known to exhibit sex differences in glucose levels (Kubota et al., 2000; Masaki et al., 2004) and survival (Withers et al., 1998). A similar sex difference was also found in the HFD mice. This has two possible explanations. Firstly, the diabetes/obesity-associated bladder enlargement could be model dependent. However, that does not explain why it was observed in one but not the other study on ob/ob mice. Second, it is also possible that this difference at least partly reflects random scatter that can occur with small sample sizes (Halsey et al., 2015). While the divergent findings between the two studies in ob/ob mice would argue for the latter, a difference in bladder/body weight by about 35% is not very likely to occur based on chance alone.

### 4.4 mRNA expression differences in diabetic/obese mice

An exploratory comparison of mRNA expression for genes proposed to be involved in the pathophysiology of bladder enlargement or in the fibrosis/inflammation pathway was a preplanned secondary outcome assessment of our study, which for practical reasons (amount of available tissue) was limited to three of the studies reported here. Regarding pathophysiology, we have analyzed expression of NGF and two subtypes of angiotensin II receptors AT_1_ and AT_2_; the originally planned analysis of expression of the NGF receptor trk A did not yield usable results because expression level was too low to enable meaningful quantification. While mRNA expression of the AT_1_ receptor was about 30% greater in the pooled control groups of female than male mice, the CI was wide. On the other hand, expression of NGF and AT_2_ receptors was similar in both sexes. Similarly, our findings for the three parameters related to fibrosis (collagen I and III and TGF-β) on to MCP-1 as parameter of inflammation exhibited numeric differences that could be of biological relevance but in most cases had CI that were too wide for a robust conclusion. While the sample sizes of 17–19 mice per group are large compared to that in many other studies reporting on mRNA expression of such parameters, our data can neither robustly demonstrate nor rule out such differences due to the large variability within each group. Studies within the control group or within the diabetic/obese group of each study had similar variability but smaller sample sizes. Therefore, we report those only for information but do not draw conclusions based on these data. Thus, these data do not robustly support the idea of sex differences for any of these seven parameters but unfortunately are insufficient to exclude a biologically relevant difference.

## 5 Conclusion

In conclusion, our findings are validated by confirming known findings such as the body weight difference in the pooled controls and the glucose level difference in IRS2 knock-out mice. Our data show an absence of sex differences in bladder/body weight ratio in healthy controls. Among the diabetic/obese groups, substantial (about 35%) differences were found in three of six comparisons but not in three others. Particularly based on the divergent findings in the ob/ob mice studies we cannot rule out that this at least partly can be related to limited sample sizes. However, our data indicate that a sex difference in bladder/body weight ratio may be model-dependent. Additional studies in hypothesis-testing mode (Vollert et al., 2022) will be required to robustly determine this.

## Data Availability

The original contributions presented in the study are included in the article/Supplementary Material, further inquiries can be directed to the corresponding author.
