# Supplementary material for: A comparison of urinary bladder weight in male and female mice across five models of diabetes and obesity
Source: Front Pharmacol. 2023 Feb 20;14:1118730. doi: 10.3389/fphar.2023.1118730 (PMC9986474; doi:10.3389/fphar.2023.1118730)

Online Supplement to

A comparison of urinary bladder weight in male and female mice across five models of diabetes and obesity

Betül R. Erdogan, Martina B. Michel, Jan Matthes, Tamara R. Castañeda, Urs Christen, Ebru Arioglu-Inan, Martin C. Michel, Andrea Pautz

Supplemental Table 1:

Primers used in current study. Primers were derived from the mouse genomic sequence (see Methods in main paper), except for β-actin which was from the rat genome sequence; "base pairs" describes the size of the expected PCR product.

gene	forward	reverse	base pairs
GAPDH	GAGAGTGTTTCCTCGTCCCG	ACTGTGCCGTTGAATTTGCC	238
β-actin	ACTGTGCCGTTGAATTTGCC	TGTGTTGGCATAGAGGTCTTTACG	145
NGF	AAAGGTTTTGCCAAGGACGC	CTGTGTACGGTTCTGCCTGT	144
trk A	GCGATGACGTGTTTCTGCAG	CCCACATCATTCTCTGCCCA	188
AT1R	ACTCACAGCAACCCTCCAAG	CCTGGGGCAGTCATCTTGAA	145
AT2R	ACTGCTTTAAACACTGGCAACT	GTGCCAGTTGCAGATT	140
collagen I	GCTCCTCTTAGGGGCCACT	CCACGTCTCACCATTGGGG	102
collagen III	CCTTCCAGGACAACCAGGTC	CGGATAGCCACCCATTCCTC	141
TGF-β1	TGAGGTCACTGGAGTTGTACGG	GGTTCATGTCATGGATGGTGC	170
MCP-1	TCCCAATGAGTAGGCTGGAG	TCTGGACCCATTCCTTCTTG	125
Mu IL-6	GAGGATACCACTCCCAACAGACC	AAGTGCATCATCGTTGTTCATACA	140

Supplemental Figure 1:

Comparison of reference gene expression between sexes. Data are means ± SD of pooled control mice from 3 studies (male: n = 18, female n = 19). Note that two values for GADPH (mice 32g and 90g, both female IRS2 knock-out animals) were removed from the analysis because the melting curves did not meet the quality criteria. Target gene expression was normalized only based on β-actin values in these cases.

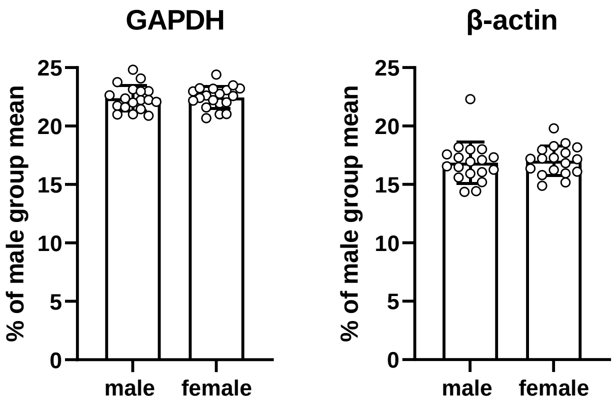

Supplemental Figure 2:

Glucose, body weight, bladder weight and BBW in the control mice of RIP-LCMV study. Data are means ± SD and each data point represents one animal. Data from the pooled control groups of all studies are shown as Figure 1 in the main paper.

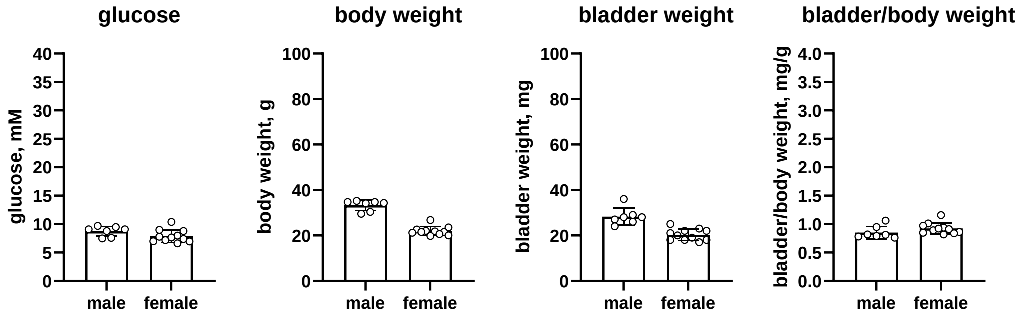

Supplemental Figure 3:

Glucose, body weight, bladder weight and BBW in the control mice of db/db and ob/ob studies (Hoechst). Data are means ± SD and each data point represents one animal. Data from the pooled control groups of all studies are shown as Figure 1 in the main paper.

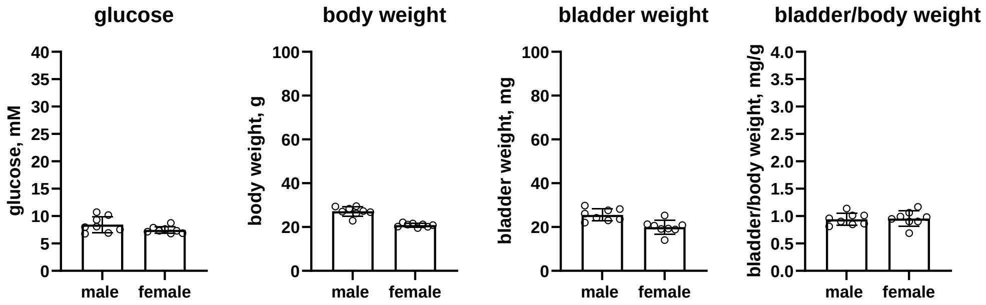

Supplemental Figure 4:

Glucose, body weight, bladder weight and BBW in the control mice of the ob/ob study (Cologne). Data are means ± SD and each data point represents one animal. Data from the pooled control groups of all studies are shown as Figure 1 in the main paper.

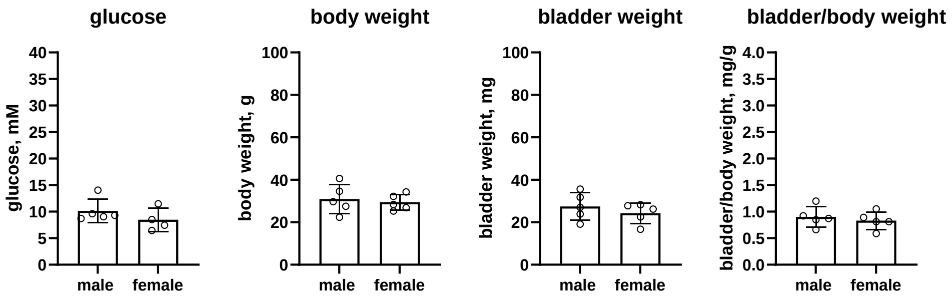

Supplemental Figure 5:

Glucose, body weight, bladder weight and BBW in the control mice of the IRS2 knock-out study. Data are means ± SD and each data point represents one animal. Data from the pooled control groups of all studies are shown as Figure 1 in the main paper.

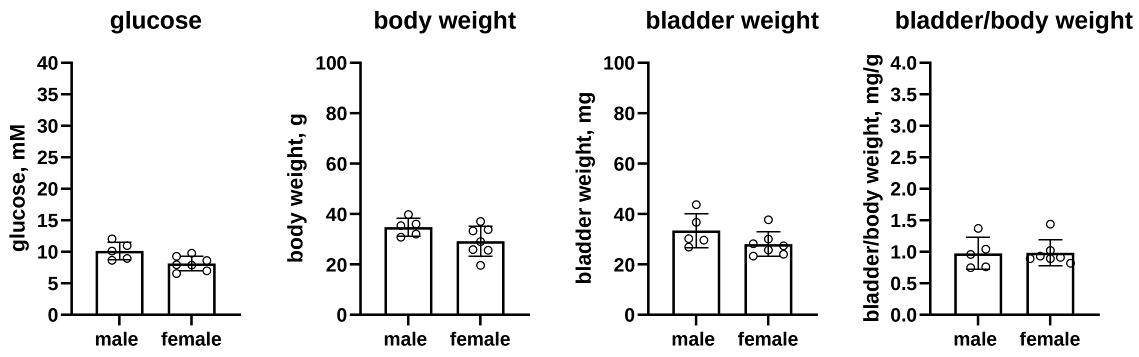

Supplemental Figure 6:

Glucose, body weight, bladder weight and BBW in the control mice of the high-fat diet study. Data are means ± SD and each data point represents one animal. Data from the pooled control groups of all studies are shown as Figure 1 in the main paper.

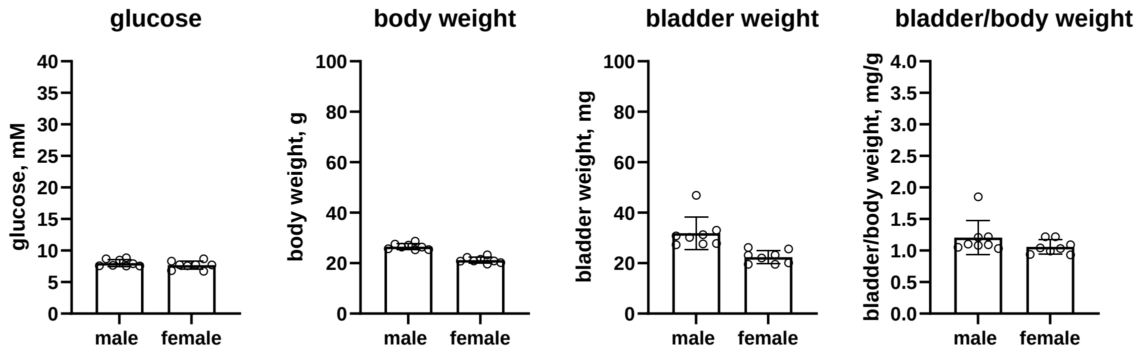

Supplemental Figure 7:

mRNA expression of NGF and the AT_1 and AT_2 receptor in control mice used for the ob/ob and db/db (Hoechst) mice. Data are means ± SD and each data point represents one animal.

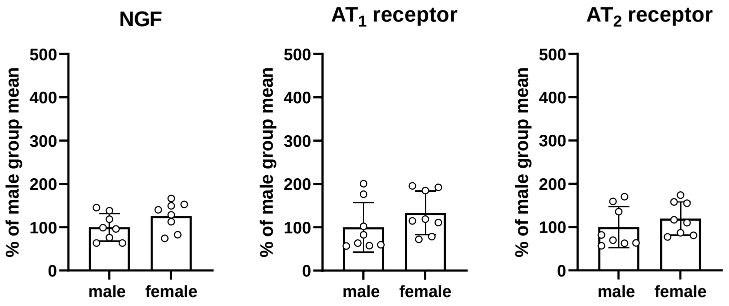

Supplemental Figure 8:

mRNA expression of collagen I and III, TGF-β and MCP-1 in control mice used for the ob/ob and db/db (Hoechst) mice. Data are means ± SD and each data point represents one animal.

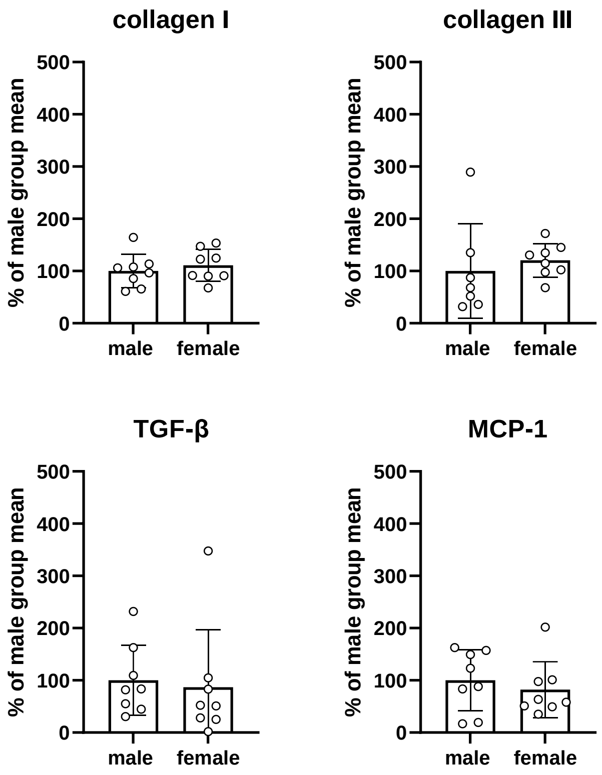

Supplemental Figure 9:

mRNA expression of NGF and the AT_1 and AT_2 receptor in db/db mice. Data are means ± SD and each data point represents one animal.

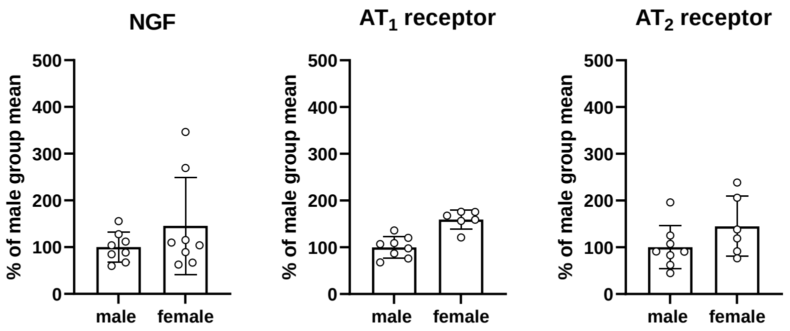

Supplemental Figure 10:

mRNA expression of collagen I and III, TGF-β and MCP-1 in db/db mice. Data are means ± SD and each data point represents one animal. Two female animals (mice 18 and 20) were removed from the analysis of TGF-β as outlier based on values of 3827% and 15,502%, respectively.

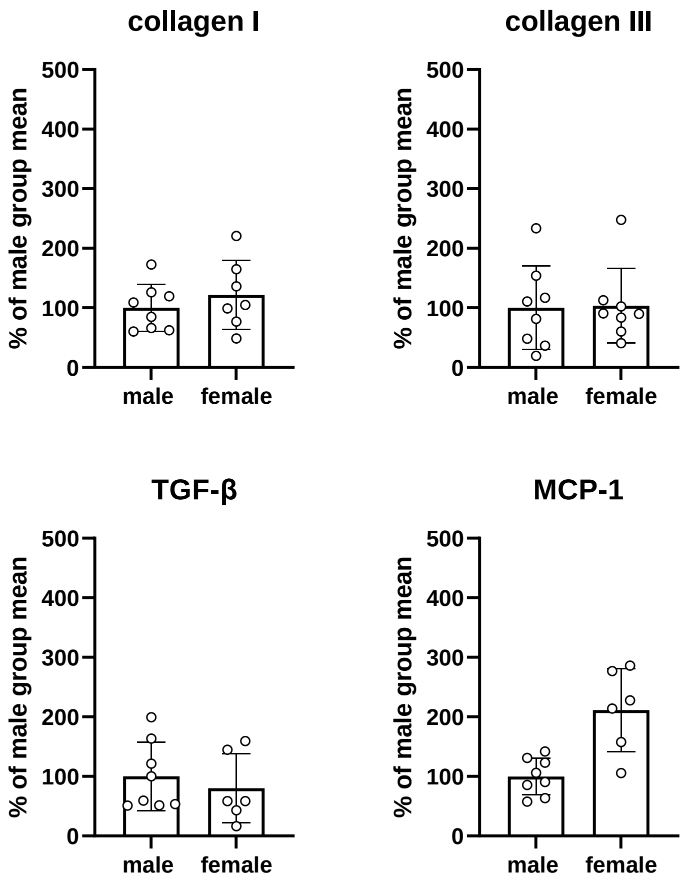

Supplemental Figure 11:

mRNA expression of NGF and the AT_1 and AT_2 receptor in ob/ob mice (Hoechst). Data are means ± SD and each data point represents one animal.

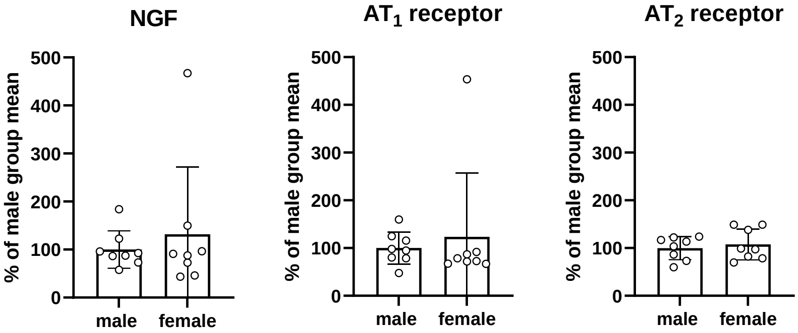

Supplemental Figure 12:

mRNA expression of collagen I and III, TGF-β and MCP-1 in ob/ob mice (Hoechst). Data are means ± SD and each data point represents one animal. One female animal (mouse 25) was removed from the analysis of MCP-1 as outlier based on a value of 1172%.

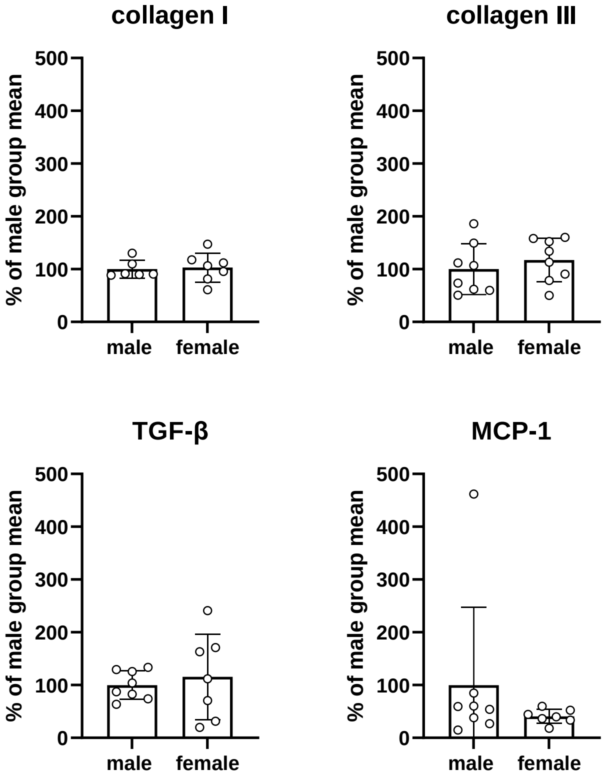

Supplemental Figure 13:

mRNA expression of NGF and the AT_1 and AT_2 receptor in control mice used for the ob/ob (Cologne) mice. Data are means ± SD and each data point represents one animal. Data from mouse 46f (female) had an outlier value for actin and were normalized only for GAPDH expression.

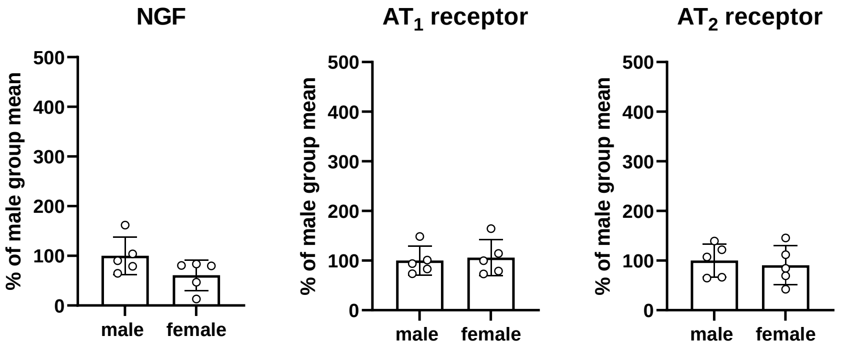

Supplemental Figure 14:

mRNA expression of collagen I and III, TGF-β and MCP-1 in control mice used for the ob/ob (Cologne) mice. Data are means ± SD and each data point represents one animal. Data from mouse 46f (female) had an outlier value for actin and were normalized only for GAPDH expression.

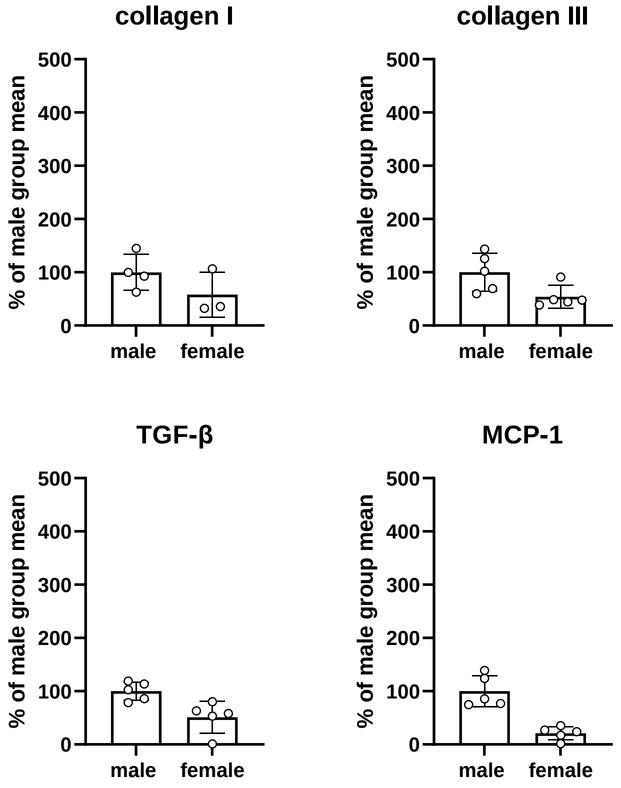

Supplemental Figure 15:

mRNA expression of NGF and the AT_1 and AT_2 receptor in ob/ob (Cologne) mice. Data are means ± SD and each data point represents one animal.

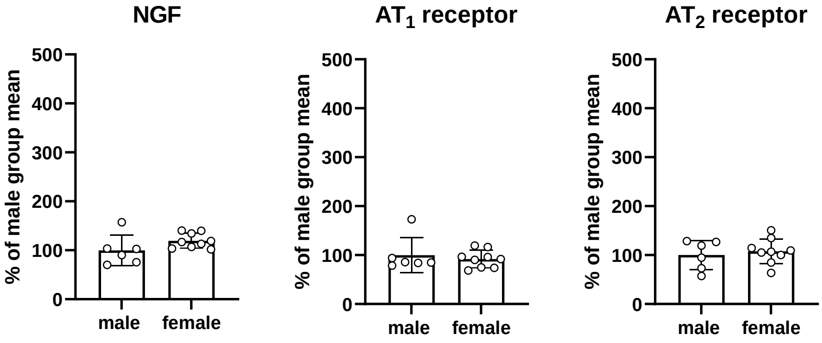

Supplemental Figure 16:

mRNA expression of collagen I and III, TGF-β and MCP-1 in ob/ob (Cologne) mice. Data are means ± SD and each data point represents one animal.

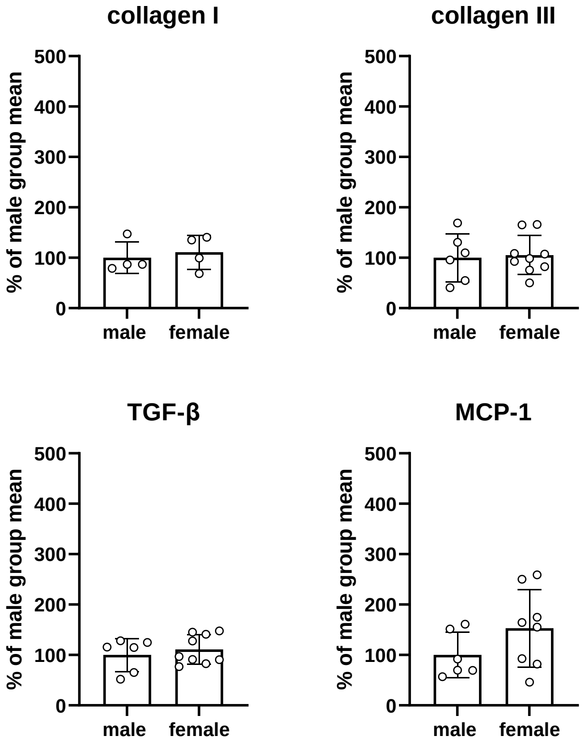

Supplemental Figure 17:

mRNA expression of NGF and the AT_1 and AT_2 receptor in control mice used for the IRS2 knock-out mice. Data are means ± SD and each data point represents one animal. Data from mouse 9g (female) were removed from the analysis because expression level was close to water control for most parameters.

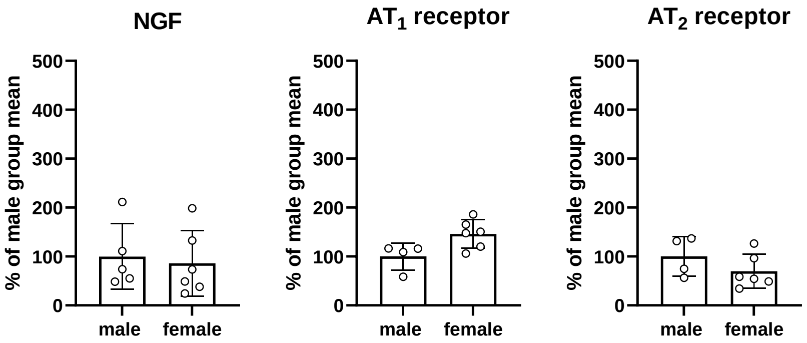

Supplemental Figure 18:

mRNA expression of collagen I and III, TGF-β and MCP-1 in control mice used for the IRS2 knock-out mice. Data are means ± SD and each data point represents one animal. Data from mouse 9g (female) were removed from the analysis because expression level was close to water control for most parameters.

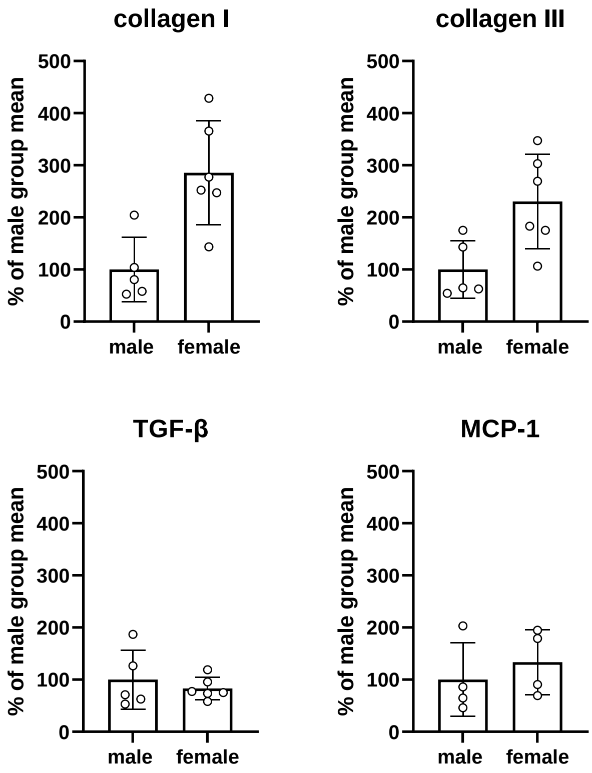

Supplemental Figure 19:

mRNA expression of NGF and the AT_1 and AT_2 receptor in IRS2 knock-out mice. Data are means ± SD and each data point represents one animal.

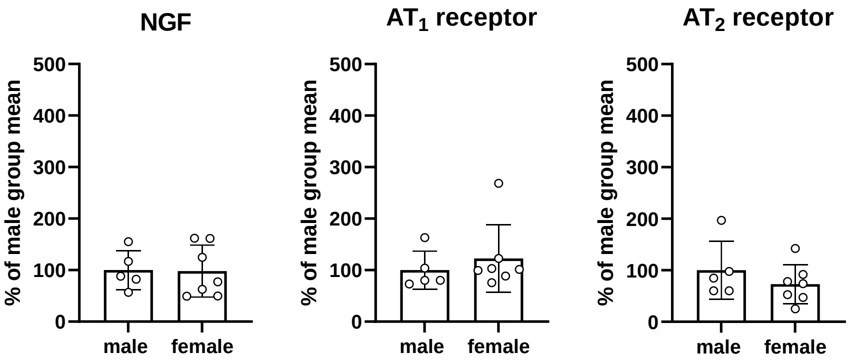

Supplemental Figure 20:

mRNA expression of collagen I and III, TGF-β and MCP-1 in IRS2 knock-out mice. Data are means ± SD and each data point represents one animal.

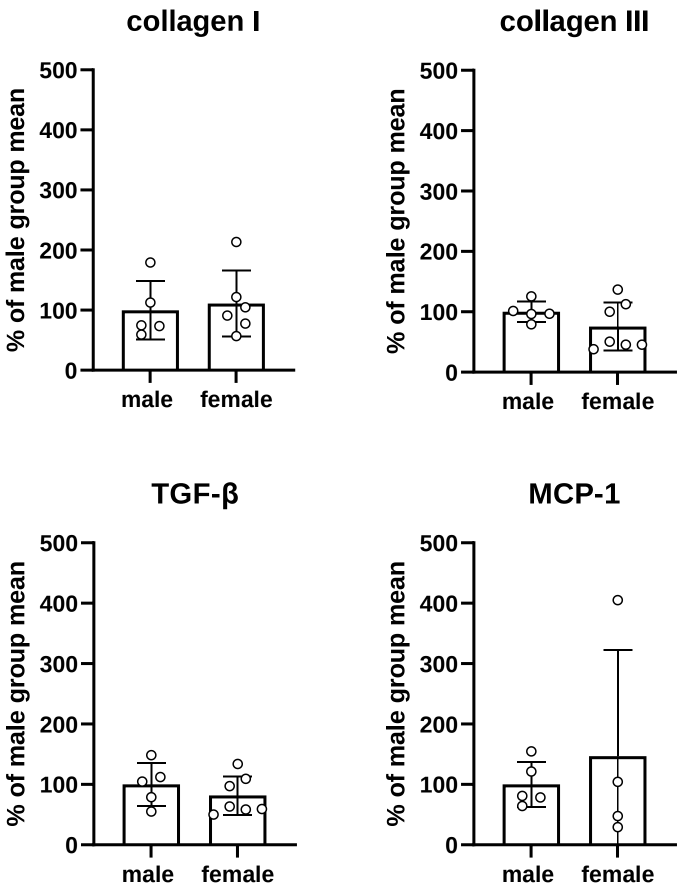

Supplement: Supplementary file 1 [file Presentation1.pdf]
